# Assessing Alternatives to Locomotion Scoring for Detecting Lameness in Dairy Cattle in Tanzania: Infrared Thermography

**DOI:** 10.3390/ani13081372

**Published:** 2023-04-17

**Authors:** Chacha W. Werema, Linda J. Laven, Kristina R. Mueller, Richard A. Laven

**Affiliations:** 1 School of Veterinary Science, Massey University, Private Bag 11 222, Palmerston North 4442, New Zealandr.laven@massey.ac.nz (R.A.L.); 2 College of Veterinary Medicine and Biomedical Sciences, Sokoine University of Agriculture, Morogoro 67 115, Tanzania

**Keywords:** lameness, locomotion scoring, infrared thermography, dairy cattle, tropical country

## Abstract

**Simple Summary:**

Locomotion scoring requires skilled, trained observers to accurately detect lameness. Many studies have thus evaluated infrared thermography as an alternative lameness detection method as it does not require a skilled observer. However, there are few reports of the use of infrared thermography in cattle in tropical environments like Tanzania. This study, therefore, aimed to assess whether using an infrared camera to measure the foot skin temperature of hind limbs could potentially be used as an alternative on Tanzanian dairy farms. Three study farms were visited twice each during the afternoon milking on consecutive days. Locomotion scoring using a 4-point scale (0–3) was conducted on the first day as the cows exited the milking parlour after being milked. On the following day, the hind limbs of the cows were thermally imaged while they were standing in the milking parlour, using a forward-looking infrared camera. Mean foot skin temperature increase was associated with an increase in locomotion score; for example, the mean temperature was higher for cows with a locomotion score of 3 than those with a score of 2. Therefore, the present study confirmed that measuring foot skin temperature using an infrared camera has the potential to be employed for detecting lameness on Tanzanian dairy farms. However, improvements in accuracy and reductions in infrared camera costs are needed.

**Abstract:**

Lameness detection is a significant challenge. Locomotion scoring (LS), the most widely used system for detecting lameness, has several limitations, including its subjective nature and the existence of multiple systems, each with its own advantages and disadvantages. Therefore, this study aimed to evaluate whether the foot skin temperature (FST) of hind limbs, as measured using infrared thermography (IRT), could potentially be used as an alternative on Tanzanian dairy farms. Each of the three study farms were visited twice during the afternoon milking on consecutive days, with a total of 170 cows assessed. DairyNZ LS (4-point scale (0–3)) was undertaken on the first day as the cows exited the milking parlour after being milked, while on the following day, the plantar aspect of the hind limbs of the cows was thermally imaged while they were standing in the milking parlour, using a handheld T650sc forward-looking infrared camera. Mean FST was higher for cows with a locomotion score of 1 than those with a score of 0; higher for cows with a locomotion score of 2 than those with a score of 1; and higher for cows with a locomotion score of 3 than those with a score of 2, with each one-unit locomotion score increase being associated with a 0.57 °C increase in mean temperature across all zones. The optimal cut-off point of 38.0 °C for mean temperature across all zones was identified using a receiver operator characteristic curve. This cut-off point had a sensitivity of 73.2% and a specificity of 86.0% for distinguishing cows with a locomotion score ≥ 2 (clinical lameness). The prevalence of clinical lameness across all three farms was 33%, which meant that only 72% of cows with a mean FST across all zones ≥ 38.0 °C had been identified as clinically lame using LS. This study confirmed that IRT has the potential to be used to detect lameness on Tanzanian dairy farms. However, before it can be widely used, improvements in accuracy, especially specificity, are needed, as are reductions in equipment (IR camera) costs.

## 1. Introduction

Early lameness detection accompanied by effective treatment is crucial to minimise the pain and discomfort associated with lameness [1,2,3], as well as to decrease the risk of irreversible claw damage [4].

Visual locomotion scoring (LS) is the most commonly used active lameness detection method on dairy farms [5]. A wide range of different systems have been used; Schlageter-Tello et al. [5] identified 25 different LS systems that had been published in the peer-reviewed literature. Across all systems, the most critical challenge of LS systems is their subjective nature, with both within- and between-observer variation being high, especially when training is limited [6,7,8,9]. Thus, training is particularly important when farmers and farm staff are undertaking lameness detection using LS.

An objective technique for identifying lame cows would thus be useful for on-farm detection of lameness, especially where farm staff training is difficult to achieve (e.g., because of lack of infrastructure or due to limited farmer knowledge). This is the situation in Tanzania, where active lameness detection using LS is extremely rare. In Tanzania, farm size is generally small and infrastructure limited, making training at the farm level expensive and challenging.

One potential alternative to visual LS is infrared thermography (IRT), a non-invasive technique that records body surface temperature and produces a pictographic representation of the scanned anatomical area [10]. Body extremities and surface temperature mainly depend on blood perfusion and tissue metabolism [11]. Thus, changes in blood flow can influence the amount of radiated heat and can thus be detected by IRT [10]. One reason for changes in tissue blood flow is the inflammatory response. Thus, IRT has the potential to detect systemic infectious lameness such as foot-and-mouth disease [12], and localised infectious lameness such as bovine digital dermatitis [13,14,15], as well as non-infectious claw horn disease [16,17,18].

However, hoof temperature can be affected by factors other than foot diseases, such as physiological status [19,20], environmental factors [21,22], and activity level [23]. Thus, the production system and management of the cows in a herd can significantly affect hoof temperature, potentially altering the accuracy of IRT as a means of lameness detection. Thus to determine the usefulness of IRT in a system, IRT needs to be tested in that system [24]. Unfortunately, no data are available for tropical pastoral systems such as those which are common in many regions of Tanzania. Therefore, the aim of this study was to investigate the association between the foot skin temperature (FST) of hind limbs at afternoon milking and locomotion scoring on three Tanzanian dairy farms where cows grazed tropical pasture between morning and afternoon milking.

## 2. Materials and Methods

### 2.1. Study Area and Animals

This study was undertaken in the Morogoro region of eastern Tanzania. This region has a sub-humid tropical climate with two wet seasons per year (long rains from March to June and short rains from October to December). Mean temperature ranges from 27 to 33.7 °C and 14.2 to 21.7 °C during the dry and wet seasons, respectively.

A convenient selection of three dairy farms was made for this study. All three herds had >50 cows, were milked twice daily, grazed natural pasture off-farm between morning and afternoon milking, and had a flat concrete surface outside the milking parlour which was suitable for locomotion scoring. Calving was all year round on the three study farms, and the herds were composed of mostly multiparous milking cows, as no regular replacement of cows was done on any of the farms. All three farms had only dairy breed cattle—a mixture of breeds and crossbreeds of European dairy cattle (Friesian, Ayrshire, Jersey, and their crossbreeds) and grazed their cattle on natural pastures (principally *Hyparrhenia* spp., *Megathyrsus maximus*, *Cenchrus ciliaris*, and *Brachiaria* spp.) for approximately 8 h after morning milking.

The distance between the grazing area and milking parlour on all farms was approximately 5 to 10 km with no constructed trackways. After the afternoon milking session, cows were allowed to graze around the farm before being taken to the free stall barns in the evening (6 pm), where they were given hay (principally, from *Chloris gayana* and *Pennisetum purpureum*) and homemade concentrates. All three farms had herringbone milking parlours. Farms 1 and 2 used milking machines (reverting to hand milking when there was no power), while farm 3 employed hand-milking only. During the study period, the milking herds had 52, 58, and 60 cows on farms 1, 2, and 3, respectively.

Routine hoof trimming and systematic locomotion scoring were not undertaken on any of the farms, and none of the farms had accurate lameness treatment records.

### 2.2. Study Visits

In March 2020, farms were visited during the afternoon milking on two consecutive days. Locomotion scoring was undertaken on the first day, and on the second day the plantar aspects of both hind feet of all cows were imaged using an infrared camera.

### 2.3. Locomotion Scoring

All locomotion scoring was undertaken by the first author (CWW) who was trained in the DairyNZ lameness score using a combination of video and supervised scoring of live animals (see Werema et al. [24] for further details). Prior to the study commencing in March 2020, CWW rewatched the training videos as a refresher [25,26].

On each farm, all milking cows were locomotion scored and their ear tag number recorded as they exited the milking parlour. The site chosen for scoring observations on each farm was a flat well-maintained concrete surface that was at least 25 m long, and which was cleaned after every milking session. This area allowed the observer to view at a distance so as not to disturb cow flow and locomotion.

### 2.4. Infrared Thermography

During the afternoon milking following the LS, IRT imaging was performed using a handheld T650sc Forward-looking Infrared camera (FLIR Systems, Oregon, United States) that at foot skin temperatures had an accuracy of ±0.4 °C. Emissivity was set at 0.95. Pictures were taken while the observer was standing at a distance of approximately 1 m from the cow, with the plantar aspects of both hind feet being imaged. Prior to imaging, no foot preparation was undertaken, except that the cows walked through a footbath containing only water on entering the collecting yard for the milking parlour approximately 30 min before milking.

**Figure 1:**
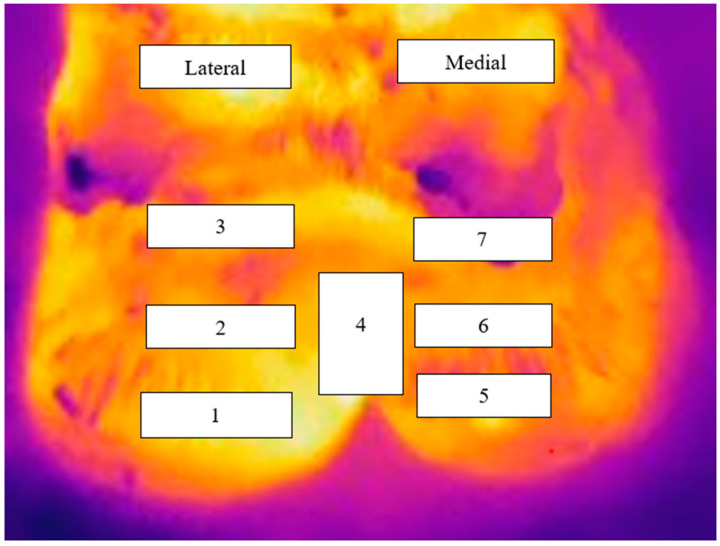
Infrared thermography image of the plantar aspect of the left hind foot overlaid to depict the seven zones for which estimates of surface skin temperature were measured. On the lateral claw; zone 1: coronary band (CB), zone 2: above the coronary band (ACB), zone 3: below accessory digit (BAD), zone 4: interdigital space (IDS). Zones 5 to 7 are the equivalent to zones 1 to 3 but on the medial claw.

Foot images were analysed using FLIR Tools software (FLIR Systems Oregon, United States) with estimates of the surface temperature obtained from seven zones on each hind foot as described by Werema et al. [24] (see Figure 1). The maximum temperature for each zone was used for analysis in line with previous infrared studies aimed at lameness detection in the cow [24,27,28]. Mean ambient temperature during this study was 29.4 °C.

### 2.5. Statistical Data Analyses

SPSS version 27 (IBM Corporation, Armonk, NY, USA) was used for all data analysis unless otherwise reported. Descriptive statistics were created for each zone temperature measure. The normality of foot temperature was visually assessed using Q-Q plots and histograms, followed by checking the skewness and kurtosis statistics. A generalised linear marginal repeated measures model was then used to evaluate the effect of the farm, foot, and zone within the foot on skin temperature. Farm and foot (right or left hind) were the independent variables, zone within foot the repeated variable and skin temperature the outcome variable. Interaction between farm and foot was tested and removed from the final model as it was non-significant (*p* > 0.05). Covariance structure was identified using Quasi-likelihood under the independence model criterion (QIC). Residuals were checked for normality using Q-Q plots and histograms. Post-hoc pairwise comparisons between zones were undertaken using the Šidák correction for multiple comparisons [29].

The association between locomotion score and foot temperature was tested using six temperature measures (see Table 1 for temperature definitions). Univariable analyses were first performed to assess the association between the outcome (foot temperature) and predictor (locomotion score) variables. This analysis identified significant heteroscedasticity when we compared foot temperature across locomotion scores. Therefore, we employed a generalised linear model with an identity link function and robust estimators [30] to analyse the association between foot skin temperature and locomotion score. In model fitting, each temperature definition was used as the outcome variable, with farm and locomotion scores as the predictor variables. Two-way interactions between farm and locomotion scores were added, and backwards selection was then used to remove any interactions where *p* > 0.05. Both main effects were kept in the final model irrespective of *p*-value.

Six receiver operator characteristic (ROC) curves were created, one for each temperature definition, with categorised locomotion score (lame (locomotion score ≥ 2) vs. not lame (locomotion score < 2)) to establish the sensitivity and specificity of IRT to predict locomotion score ≥ 2. Area under the curve (AUC) and coordinates of the curve (CC) were then used to evaluate a model’s predictive accuracy. Optimal temperature cut-off values were identified by maximising sensitivity plus specificity. The statistical package MedCalc Version 19.5.1 (MedCalc Software, Ostend, Belgium) was then employed to calculate positive and negative predictive values for those optimal cut-off points.

## 3. Results

All milking cows were locomotion scored on all three farms. The distribution of locomotion scores is summarised in Table 2. Across the three farms, 33% of cows were identified as being lame (locomotion score ≥ 2).

### 3.1. Effect of Foot and Foot Zone on Skin Temperature

On average the FST of the left foot was higher than the right foot (37.510 °C and 37.411 °C, respectively). However, it is unlikely that this is biologically significant as mean difference was only 0.099 °C (95% CI: 0.003–0.194). However, biologically significant differences between zones were identified. For example, the difference between the zone with the lowest mean temperature (zone 6) and the zone with the highest mean temperature (zone 4) was 1.57 °C (95% CI: 1.392–1.748). Results for all zones and their comparisons are summarised in Table 3 and Table 4, respectively. Mean temperatures were higher for zones on the lateral claw than their equivalent zones on the medial claw (see Table 3); these differences were 0.243 °C, 0.284 °C, and 0.163 °C for zones 1 vs. 5, 2 vs. 6, and 3 vs. 7, respectively.

#### 3.1.1. Infrared Thermography versus Locomotion Scoring

The mean FST for different locomotion scores is summarised in Table 5. For all six FST measures (see Table 1 for definitions), the temperature was higher for cows with a locomotion score of 1 than those with a score of 0; higher for cows with a locomotion score of 2 than those with a score of 1; and higher for cows with a locomotion score of 3 than those with a score of 2. The comparisons of all six-foot skin temperature measures versus different locomotion scores are presented in Table 6. As an example, for mean temperature, the mean difference between cows with scores 0 and 1 was 0.91 °C (95% CI: 0.498–1.313); between scores 1 and 2 cows, it was 0.83 °C (95% CI: 0.431–1.232); and between scores 2 and 3 cows, it was 0.65 °C (95% CI: 0.026–1.271).

#### 3.1.2. Association of Foot Temperatures and Locomotion Scores

A linear association was demonstrated between individual cow locomotion scores and foot skin temperature for all six temperature measures. Detailed data are presented in Table 7. As an example, for mean temperature, each one-unit locomotion score increase was associated with a 0.57 °C (95% CI: 0.46–0.70) rise in mean temperature.

#### 3.1.3. A Receiver Operating Characteristic (ROC) Analysis

**Figure 2:**
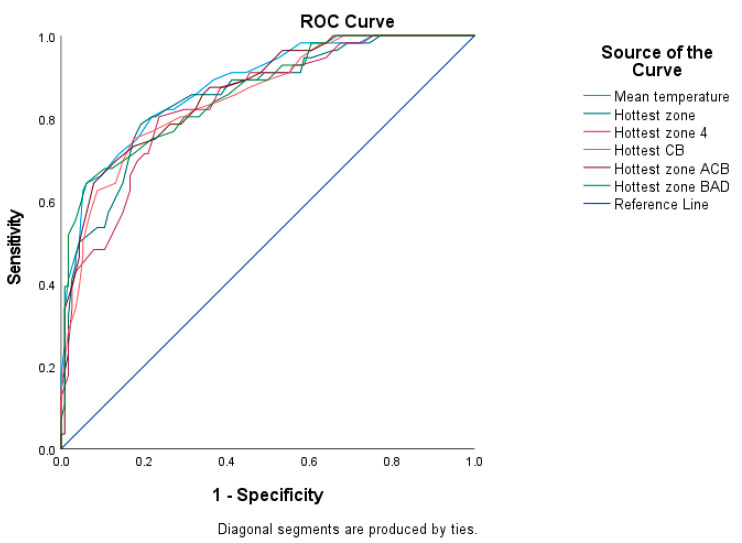
Receiver operating characteristic (ROC) curves were used to determine the optimal threshold values for the infrared thermography’s sensitivity and specificity, assuming locomotion scores ≥ 2 as locomotion score = 2 on three dairy farms in Morogoro (*n* = 170). Note: coronary band (CB), above the coronary band (ACB), below accessory digit (BAD).

A receiver operating curve is illustrated in Figure 2. Optimal threshold values, area under the curve and calculated parameters for temperature measures are summarised in Table 8.

## 4. Discussion

The objective of the current study was to assess the usefulness of infrared thermography (IRT) as a tool for detecting lameness in dairy cattle partly grazed and housed in a tropical region (Tanzania) versus visual locomotion scoring (LS).

### 4.1. Suitability of IRT for Measuring Foot Skin Temperature during Milking

On all three farms, unlike in New Zealand [24], there was sufficient time during milking to collect thermal images from all cows. Hand milking increased the time available for image collection as it was slower than when the machine was used; but during hand milking, the milker obstructed the cow’s feet, and the cows were more restless, leading to frequent changes in foot posture. Thus, during hand milking, images were collected prior to a cow being milked.

One limitation of this study is that only hind feet were imaged, as cows were photographed during milking. Not all lameness is hind-feet related; although data from housed cows suggest that hind limb lameness accounts for >90% of cases [31], in pasture-based dairy cattle in New Zealand, only 67% of lameness was in the hind limb [32]. No such data are available from Tanzanian dairy cattle. However, it is likely that only recording the FST of hind limbs lowered the sensitivity of IRT for lameness detection in these cattle. Further research is required to identify how much the sensitivity was lowered.

Another potential issue with IRT under Tanzanian conditions is the imaging of dirty feet. Prior to milking on all three farms, the cows were walked through a footbath containing water. This was routine practice on all three farms and was undertaken with the intention of reducing dirt on feet, although it is unlikely that there was any impact on foot cleanliness [33]. Although this study was undertaken in the dry season, the feet of all cows were generally clean and were not washed prior to IRT. In the rainy season, the accumulation of mud on the feet during grazing might influence the accuracy of IRT, especially if the feet are not cleaned before imaging. However, Stokes et al. [27] reported that in the UK, maximum sensitivity plus specificity of IRT was achieved in feet that were not cleaned. Further longitudinal research is required to investigate whether this is also the case in Tanzania.

### 4.2. Effect of Claw and Zone on Skin Foot Temperature

In these cattle, the lateral claw had a higher mean temperature than the medial claw, with the highest difference of 0.28 °C being between zones 2 and 6 (see Table 3). This difference is somewhat larger than the difference of 0.1 °C we found under New Zealand conditions using the same camera and protocol for IRT [24], but it is smaller than the differences of 1.0 to 1.7 °C that have been reported in studies on housed cows [17,19]. The reason for this difference is unclear, but may be related to different protocols and different equipment.

The effect of the claw was consistent across zones, with the mean temperature for zones on the lateral claw being higher than their equivalents on the medial claw (see Table 3). Additionally, the order was consistent across claws, such as if on the lateral claw, zone 1 had a higher mean temperature than zone 2, the same applied to zones 5 and 6 on the medial claw. However, the order was not the same as that reported by Werema et al. [24]. In the current study, zones 1 and 5 (coronary band) had a higher mean temperature than zones 3 and 7 (below accessory digit), whereas Werema et al. [24] reported that zones 3 and 7 had a higher temperature than zones 1 and 5. The reason for this difference is unclear. It may be related to differences in hindlimb disease (e.g., increased infectious lameness in Tanzanian cattle). However, as we did not record hoof or limb lesions in either study, this has to remain a suggestion.

### 4.3. Infrared Thermography as a Predictor of Locomotion Score

In this study, mean foot skin temperature increased as locomotion scores increased (Table 5), consistent with previous studies of IRT and locomotion scores that used different protocols and devices [24,34,35]. Consistent with Werema et al. [24], the current study found that each of the locomotion scores (0, 1 and ≥2) had significantly different mean FST. The key difference between our two studies in regard to FST and locomotion score was that in Tanzanian cattle, the optimal cut-off point was 38.0 °C, whereas in New Zealand, our optimal cut-off was 34.5 °C [24]. However, both these cut-offs are much higher than the cut-offs used in studies on housed cattle in the Northern Hemisphere, e.g., 23.3 °C [35], 25.25 °C [21], 25.5 °C, [34], and 27.0 °C [27]. The reason for the large difference in optimal cut-off between our studies and those previous studies is unclear. However, it may be related to protocol differences (e.g., measuring different sites, using different equipment or cleaning feet before imaging), differences in the environment such as ambient temperature, or differences in the cause of lameness (e.g., infectious vs. non-infectious lameness). Further data on the factors driving these differences in the optimal cut-off points are required. However, our data suggest that optimal cut-offs are likely to be protocol and production system specific. Thus, cut-offs should not be transferred from one study in one system to another, even if similar protocols are used.

The specificity and sensitivity of IRT at our 38.0 cut-off point for identifying cows with a locomotion score of ≥2 were both moderate (86.0 and 73.2%, respectively), and lower than the specificity and sensitivity we reported in New Zealand of 92.4 and 80.0%, respectively [24]. However, as with the difference between the two studies in relation to optimal cut-off, the difference between our two studies is much less than the differences between previous studies, which have reported the specificity and sensitivity of IRT. For example, Lin et al. [35] reported a sensitivity of 78.5% and specificity of 39.2%, while the equivalent figures for Rodríguez et al. [34] were 46.7% and 89.7%, respectively; and for Stokes et al. [27], they were 80% and 73%, respectively. As with optimal cut-off, we need more data on the factors which are driving this variability in specificity and sensitivity, but the much smaller differences between our two studies suggest that differences in protocols may be driving much of the difference between studies.

For alternative methods of lameness detection, it is the specificity that is the most important. If a technique is easy to apply, then moderate sensitivity can be overcome by repeated measurement. However, even relatively high specificity (>90%) can result in farmer fatigue if a high proportion of identified cows are not actually lame [36]. This fatigue is determined by the positive predictive value (PPV), the proportion of the positive tests which are true positive. The PPV is determined by specificity and prevalence; thus, although the specificity was lower in our Tanzanian study than in New Zealand, the much higher lameness prevalence (33 vs. 14%) meant that PPV was higher in the Tanzanian study.

Nevertheless, a PPV of 72% at the optimal cut-off of 38.0 °C is not ideal as ~1/4 of the cattle identified will not have changes in gait and posture associated with clinical lameness. However, such issues may be tolerated in the small dairy herds in Tanzania, where the number of false positive cattle identified at any one time may be relatively small. Further research is required to better understand Tanzanian dairy farmers’ approach to lameness and whether this technological approach is likely to be acceptable on Tanzanian dairy farms.

One fundamental limitation of the current study is equipment cost. The FLIR camera is not likely to be affordable for most farmers, especially in lower-middle-income countries like Tanzania. However, the costs of infrared cameras are decreasing, and the development of smartphone apps which utilise IRT technology may make the technology affordable, even for Tanzanian farmers. Thus, our data suggest that if these changes continue, IRT may, in the future, be a useful objective method of detecting lameness in Tanzanian dairy cows.

## 5. Conclusions

The current study showed that, with a trained observer, locomotion scoring after milking can be a useful method of lameness detection under Tanzania conditions. However, the study farms were chosen based on having a suitable area for observation, so may not be typical of Tanzanian dairy farms. Further evaluation of LS on Tanzanian dairy farms is warranted, but the requirement for a trained observer may remain a key issue. The present study results demonstrated that FST measured by IRT was able to differentiate between cows with different locomotion scores. However, the sensitivity, and especially the specificity of IRT need to be improved before it can be recommended for lameness detection under Tanzanian conditions. Additionally, the development of cheap, accurate smartphone IRT apps is needed for such technology to become affordable even on large Tanzanian dairy farms.

## Figures and Tables

**Table 1:** Definitions of the infrared thermography estimates utilised for analysis adopted from [24].

Foot/Zone *	Description
Mean temperature	Average temperature, across both feet (all 14 zones)
Hottest zone	Highest zone temperature, across both feet (all 14 zones)
Hottest zone 4	The highest zone 4 temperature on either foot
Hottest coronary band (CB)	The highest zone 1 or 5 temperature on either foot
Hottest above the coronary band zone (ACB)	The highest zone 2 or 6 temperature on either foot
Hottest zone below the accessory digit (BAD)	The highest zone 3 or 7 temperature on either foot

* For all analyses, the maximum temperature for each zone was used for the analysis.

**Table 2:** Distribution of locomotion score and their percentage in brackets on three farms in Morogoro, Tanzania (*n* = 170).

Farm Name	Locomotion Score				
	0	1	2	3	Total
1	11 (21.2)	24 (46.1)	12 (23.1)	5 (9.6)	52
2	15 (25.9)	25 (43.1)	15 (25.9)	3 (5.1)	58
3	13 (21.7)	26 (43.3)	14 (23.3)	7 (1.7)	60
Total	39 (23.0)	75 (44.1)	41 (24.1)	15 (8.8)	170

**Table 3:** The mean temperature (degrees centigrade) and 95% confidence interval for zones 1–7 (see Table 1 for definitions) for both hindlimbs on three dairy farms in Morogoro (*n* = 170).

Zone	Mean	95% Confidence Interval	
		Lower	Upper
1	37.82	37.78	37.87
2	37.02	36.96	37.09
3	37.46	37.45	37.47
4	38.31	38.29	38.32
5	37.58	37.52	37.65
6	36.74	36.70	36.77
7	37.30	37.26	37.33

Note: zones 1–3 are on the lateral claw, and 5–7 are equivalents on the medial claw.

**Table 4:** Comparisons of mean foot skin temperature (degrees centigrade) and 95% confidence interval for zones 1–7 (see Table 1 for definitions) for both hindlimbs on three dairy farms in Morogoro (*n* = 170).

(I) Zone	(J) Zone	Mean Difference (I − J)	95% Confidence Interval	
			Lower Bound	Upper Bound
1	2	0.80	0.53	1.08
	3	0.36	0.09	0.64
	5	0.24	−0.03	0.52
	6	1.09	0.81	1.36
	7	0.53	0.25	0.80
2	6	0.28	0.01	0.56
3	2	0.44	0.17	0.72
	6	0.72	0.45	1.00
	7	0.16	−0.11	0.44
4	1	0.48	0.21	0.76
	2	1.29	1.01	1.56
	3	0.85	0.57	1.12
	5	0.73	0.45	1.00
	6	1.57	1.30	1.85
	7	1.01	0.74	1.30
5	2	0.56	0.29	0.83
	3	0.12	−0.16	0.40
	6	0.84	0.57	1.12
	7	0.28	0.01	0.56
7	2	0.28	0.00	0.55
	6	0.56	0.29	0.84

**Table 5:** The mean temperature (degrees centigrade) from plantar aspects across both feet (all 14 zones) and 95% confidence interval for different locomotion scores (0–3) DairyNZ on three farms in Morogoro (*n* = 170).

Locomotion Score	Mean	95% Confidence Interval	
		Lower	Upper
0	36.45	36.12	36.76
1	37.35	37.19	37.51
2	38.19	38.00	38.38
3	38.81	38.63	38.99

**Table 6:** Comparisons of mean foot skin temperature for different zones versus locomotion scores (0–3) DairyNZ on three dairy farms in Morogoro (*n* = 170).

Temperature Measure	(I) LS	(J) LS	Mean Difference (I − J)	95% Confidence Interval	
				Lower Bound	Upper Bound
MT	1	0	0.91	0.50	1.31
	2	0	1.74	1.28	2.20
		1	0.83	0.43	1.23
	3	0	2.39	1.76	3.01
		1	1.48	0.90	2.06
		2	0.65	0.03	1.27
Hottest zone	1	0	0.83	0.42	1.24
	2	0	1.56	1.10	2.03
		1	0.73	0.33	1.13
	3	0	2.26	1.63	2.90
		1	1.43	0.84	2.02
		2	0.70	0.07	1.33
Hottest zone 4	1	0	0.89	0.44	1.35
	2	0	1.57	1.06	2.09
		1	0.68	0.23	1.12
	3	0	2.36	1.66	3.06
		1	1.47	0.82	2.11
		2	0.79	0.09	1.48
Hottest CB	1	0	0.81	0.37	1.25
	2	0	1.60	1.10	2.09
		1	0.79	0.36	1.22
	3	0	2.33	1.65	3.00
		1	1.52	0.89	2.14
		2	0.73	0.06	1.40
Hottest zone ACB	1	0	0.82	0.38	1.26
	2	0	1.68	1.18	2.19
		1	0.86	0.43	1.30
	3	0	2.26	1.58	2.94
		1	1.44	0.80	2.07
		2	0.57	−0.10	1.25
Hottest zone BAD	1	0	0.87	0.47	1.27
	2	0	1.64	1.18	2.10
		1	0.77	0.37	1.17
	3	0	2.36	1.74	2.98
		1	1.49	0.91	2.07
		2	0.72	0.11	1.34

MT = mean temperature, CB = coronary band, ACB = above the coronary band, BAD = below the accessory digits.

**Table 7:** Temperature measures estimates and their 95% confidence intervals on three dairy farms in Morogoro (*n* = 170).

Model Parameter		95% Confidence Interval	
		Lower Bound	Upper Bound
Mean temperature (intercept)	38.66	38.39	38.93
Locomotion score *	0.57	0.46	0.70
Hottest zone (intercept)	40.06	39.81	40.31
Locomotion score *	0.57	0.46	0.70
Hottest zone 4 (intercept)	39.81	39.56	40.07
Locomotion score *	0.70	0.57	0.87
Hottest coronary band (intercept)	39.47	39.18	39.75
Locomotion score *	0.66	0.53	0.81
Hottest zone ACB (intercept)	38.47	38.18	38.76
Locomotion score *	0.67	0.54	0.83
Hottest zone BAD (intercept)	39.13	38.90	39.37
Locomotion score *	0.56	0.46	0.70

* Effect of increased locomotion score of 1 unit in DairyNZ lameness score. See Table 1 for the definition of temperature measurement. ACB = above the coronary band, BAD = below the accessory digits.

**Table 8:** Optimal cut-off points for skin foot temperature measurements (degrees centigrade) for determining lame cows (Dairy NZ lameness score ≥ 2) on three dairy farms in Morogoro (*n* = 170).

Temperature Measure ^1^	Optimal Threshold (°C)	AUC (95% CI)	Specificity (95% CI)	Sensitivity (95% CI)	PPV (95% CI) *	NPV (95% CI) *
Mean temperature	38.0	0.88 (0.83–0.93)	86.0 (78.2–91.8)	73.2 (59.7–84.2)	71.9 (61.3–80.6)	86.7 (80.8–91.0)
Hottest zone	39.0	0.85 (0.79–0.91)	80.7 (72.3–87.5)	78.6 (65.6–88.4)	66.7 (57.3–74.9)	88.5 (82.2–92.7)
Hottest zone 4	38.9	0.84 (0.78–0.90)	79.8 (71.3–86.8)	71.4 (57.8–82.7)	63.5 (53.8–72.2)	85.1 (78.8–89.7)
Hottest CB	38.6	0.85 (0.79–0.91)	82.5 (74.2–88.9)	75.0 (61.6–85.6)	67.7 (57.8–76.3)	87.0 (80.9–91.4)
Hottest zone ACB	38.1	0.87 (0.81–0.92)	86.8 (79.2–92.4)	69.6 (55.9–81.2)	72.2 (61.1–81.1)	85.3 (79.6–89.7)
Hottest zone BAD	38.7	0.86 (0.80–0.92)	93.9 (87.8–97.5)	64.3 (50.4–76.6)	83.7 (71.0–91.5)	84.3 (79.0–88.4)

^1^ See Table 1 for the definition of temperature measure; AUC, area under the curve; CI, confidence interval; PPV, positive predictive value and NPV, negative predictive value; CB, coronary band; ACB, above the coronary band; BAD, below accessory digit (BAD). *, PPV and NPV calculated at a prevalence of lameness of 32.94%.

## Data Availability

Data are available at request from the corresponding author.
